# Efficient iPS Cell Production with the MyoD Transactivation Domain in Serum-Free Culture

**DOI:** 10.1371/journal.pone.0034149

**Published:** 2012-03-30

**Authors:** Hiroyuki Hirai, Nobuko Katoku-Kikyo, Peter Karian, Meri Firpo, Nobuaki Kikyo

**Affiliations:** 1 Stem Cell Institute, Department of Medicine, University of Minnesota, Minneapolis, Minnesota, United States of America; 2 Division of Hematology, Oncology and Transplantation, Department of Medicine, University of Minnesota, Minneapolis, Minnesota, United States of America; 3 Division of Endocrinology, Department of Medicine, University of Minnesota, Minneapolis, Minnesota, United States of America; Istituto Dermopatico dell'Immacolata, Italy

## Abstract

A major difficulty of producing induced pluripotent stem cells (iPSCs) has been the low efficiency of reprogramming differentiated cells into pluripotent cells. We previously showed that 5% of mouse embryonic fibroblasts (MEFs) were reprogrammed into iPSCs when they were transduced with a fusion gene composed of *Oct4* and the transactivation domain of *MyoD* (called M_3_O), along with *Sox2*, *Klf4* and *c-Myc* (SKM). In addition, M_3_O facilitated chromatin remodeling of pluripotency genes in the majority of transduced MEFs, including cells that did not become iPSCs. These observations suggested the possibility that more than 5% of cells had acquired the ability to become iPSCs given more favorable culture conditions. Here, we raised the efficiency of making mouse iPSCs with M_3_O-SKM to 26% by culturing transduced cells at low density in serum-free culture medium. In contrast, the efficiency increased from 0.1% to only 2% with the combination of wild-type *Oct4* and SKM (OSKM) under the same culture condition. For human iPSCs, M_3_O-SKM achieved 7% efficiency under a similar serum-free culture condition, in comparison to 1% efficiency with OSKM. This study highlights the power of combining the transactivation domain of *MyoD* with a favorable culture environment.

## Introduction

iPSC technology demonstrates that differentiated cells can be dedifferentiated to a pluripotent state by introducing only a few genes, most commonly the OSKM genes [1], [2], [3]. However, protocols for generating iPSCs are inefficient and slow. Generally less than 1% of transduced cells are reprogrammed to form iPSCs with the standard OSKM transgenes. This is partly because exogenous Oct4 and Sox2 proteins cannot gain access to their target DNA sequences, perhaps due to closed chromatin structure [4]. We recently showed that a fusion gene, called M_3_O, between *Oct4* and the transactivation domain (TAD) derived from *MyoD*, a master regulator of skeletal myogenesis, reprograms 5% of MEFs into iPSCs when combined with polycistronic SKM [4], [5]. Importantly, M_3_O-SKM remodeled chromatin at the *Oct4* and *Sox2* genes to an embryonic stem cell (ESC)-like state in the majority of MEFs, including those that failed to become iPSCs, as evaluated by binding of Oct4 and Sox2 proteins and histone modification patterns at the two genes. These findings indicated that M_3_O-SKM increased the number of completed iPSCs as well as the background population of partially reprogrammed MEFs. Therefore, we speculated that if we could identify an additional cue that drives the partially reprogrammed population toward completed iPSCs, the efficiency of making iPSCs would drastically increase. The current study addressed the question of whether the culture environment could serve as the additional cue by changing the density of transduced cells and culture media.

## Results and Discussion

**Figure 1:**
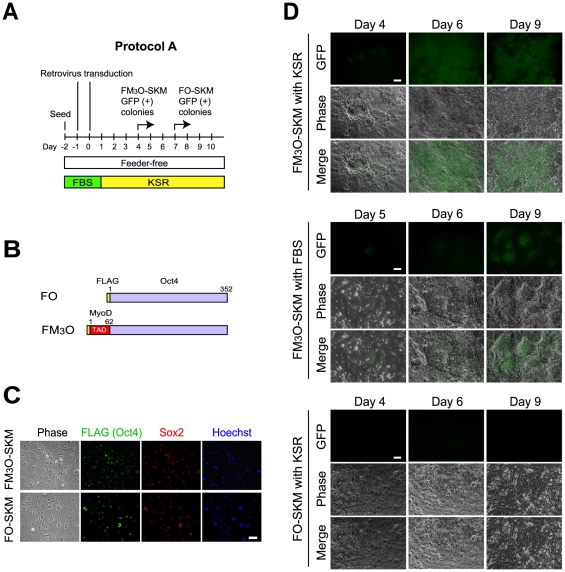
Formation of mouse iPSCs with FM_3_O-SKM and FO-SKM without subculture. (A) Schematic drawing of Protocol A. (B) Schematic drawings of FLAG-tagged Oct4 and FLAG-tagged M_3_O. Numbers indicate amino acid positions of Oct4 and MyoD. (C) Immunofluorescence staining of transduced FM_3_O, FO and Sox2. MEFs were double-immunofluorescence stained with anti-FLAG antibody, which recognized FM_3_O and FO, and anti-Sox2 antibody on day 2. DNA was counterstained with Hoechst 33342. Bar, 100 µm. (D) Appearance and increase of GFP-positive colonies after transduction with FM_3_O-SKM and FO-SKM into MEFs. While iPSC medium containing KSR was used in the top and bottom sets, KSR was replaced with 15% FBS in the middle set. Large GFP-positive colonies had fused as early as day 6 with FM_3_O-SKM and KSR as shown in the top set; however, GFP-positive areas generally diminished by day 9 when these tightly packed cells were left without subculture. Although less than 10 GFP-positive colonies appeared by day 9 with FO-SKM and KSR in a well of a 12-well plate, the majority of the areas did not contain GFP-positive colonies as shown in the bottom set. Bar, 100 µm.

We previously used feeder-free and feeder-plus protocols to make iPSCs with M_3_O-SKM [4]. With the feeder-free protocol, 3.6% of MEFs transduced with M_3_O-SKM were reprogrammed into iPSCs in a culture medium that contained 15% fetal bovine serum (FBS). In the present study, we replaced FBS with 20% KnockOut Serum Replacement (KSR), which is optimized for maintenance of ESCs in an undifferentiated state, although its contents have not been disclosed (Fig. 1A, Protocol A). KSR has also been reported to be more effective in making iPSCs than FBS [6]. In addition, we added the FLAG sequence to the amino terminus of M_3_O to create the FM_3_O construct (Fig. 1B). An anti-FLAG antibody was used to detect the protein with immunofluorescence staining because commercially available Oct4 antibodies recognized M_3_O only if the protein was denatured. The FLAG sequence did not significantly affect the efficiency of making iPSCs. The FLAG sequence was also attached to *Oct4* to prepare FO as a control. The co-transduction efficiency of FM_3_O with SKM and FO with SKM was 87.5±3.2% and 89.2±2.3%, respectively (Fig. 1C). iPSC formation was monitored by emergence of ESC-like colonies expressing the Oct4-driven GFP transgene as previously described [4]. GFP-positive colonies first appeared around day 4 with FM_3_O-SKM, and many more colonies arose over the next two days with KSR compared to FBS (Fig. 1D, top and middle). Colony formation with FM_3_O-SKM was rapid and highly efficient with KSR, leading to colony fusion and making it impossible to accurately count colonies. In contrast, colony formation with FO-SKM and KSR was late (day 7) and inefficient, with less than 0.1% of MEFs becoming iPSCs by day 10 (Fig. 1D, bottom).

**Figure 2:**
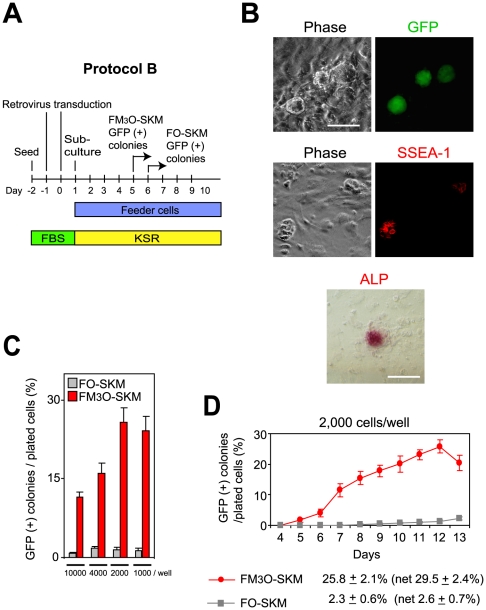
Formation of mouse iPSCs with FM_3_O-SKM and FO-SKM with subculture onto feeder cells. (A) Schematic drawing of Protocol B. (B) Expression of GFP and staining of SSEA-1 and alkaline phosphatase in iPSC colonies on day 5. Bar, 100 µm. (C) Efficiency of making iPSCs with Protocol B at different cell densities. The number of MEFs seeded on feeder cells in a 12-well plate on day 1 is written on the x axis. (D) Formation of iPSCs from MEFs seeded at 2,000 cells/well of a 12-well plate by using FM_3_O-SKM and FO-SKM in Protocol B. Mean ± SEM from three independent experiments is shown. Maximum efficiency obtained with each gene combination is shown at the bottom. In addition, efficiency was calibrated on the basis of viral transduction efficiency and described as net efficiency in parentheses.

To obtain accurate colony numbers, MEFs were seeded at various lower densities on feeder cells in the presence of KSR (Fig. 2A, Protocol B). Colonies that emerged on day 5 with FM_3_O-SKM were positive for GFP and the relatively specific pluripotency markers SSEA-1 and alkaline phosphatase (Fig. 2B). This study indicated that seeding transduced MEFs at 2,000 cells/well of a 12-well plate was most effective in making iPSCs (Fig. 2C). At this cell density, the number of GFP-positive colonies steadily increased over ten days, reaching a peak around day 12, when 25.8% of MEFs were reprogrammed to iPSCs with FM_3_O-SKM (Fig. 2D). In contrast, FO-SKM induced only 2.3% of cells to become iPSCs by day 13. Taking transduction efficiency into account, the net efficiency of making iPSCs was essentially unchanged at 29.5% (25.8/0.875) for FM_3_O-SKM and 2.6% (2.3/0.892) for FO-SKM (Fig. 2C). The current efficiency obtained with FM_3_O-SKM was substantially higher than the previous efficiency of 5.1%, which was obtained at seeding density of 50,000 cells/well in the presence of FBS [4]. When FBS was used at cell density of 2,000 cells/well, less than 2% of MEFs were reprogrammed to iPSCs with FM_3_O-SKM (data not shown). Thus, KSR and low cell density were the two major factors responsible for the significant improvement of iPSC production in the current study in comparison to our previous work [4]. Mechanistic analysis of the effects of cell density on reprogramming efficiency awaits further investigation.

**Figure 3:**
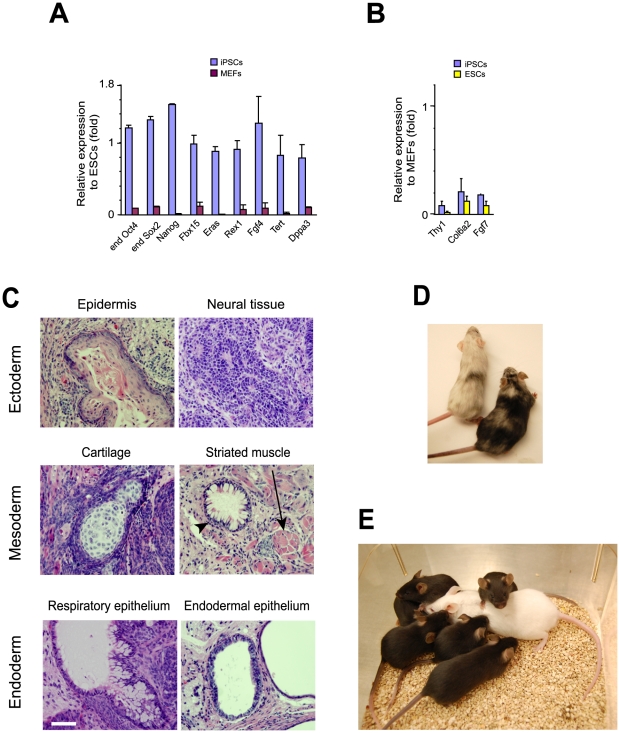
Verification of pluripotency of iPSCs prepared from MEFs with FM_3_O-SKM using Protocol B. (A) Quantitative RT-PCR analyses of pluripotency genes comparing day 10 iPSC colonies and MEFs. The value in ESCs was defined as 1.0 for each gene. Mean+SEM obtained from ten colonies is shown. (B) Quantitative RT-PCR analyses of MEF-enriched genes comparing iPSCs and ESCs. The value in MEFs was defined as 1.0 for each gene. Ten colonies were harvested on day 10. (C) Hematoxylin and eosin staining of teratoma sections derived from an iPSC clone after subcutaneous injection into an NOD/SCID mouse. The arrow and arrowhead indicate striated muscle (mesoderm) and respiratory epithelium (endoderm), respectively. Bar, 200 µm. (D) Chimeric mice prepared with an iPSC clone. High (right) and low (left) contribution of iPSCs to the skin is shown. The host 8-cell-stage embryos used to generate mice were derived from the ICR strain with a white coat color. The mottled coat color of the chimeric mice is due to the fact that iPSCs were derived from 129/B6 mice which displayed a brown or black coat color. (E) Pups obtained from mating the chimeric mouse on the right in panel D with an ICR female (white mouse shown in this photograph). This result shows that the derivatives of iPSCs contributed to germ cells.

The pluripotency of iPSCs prepared with FM_3_O-SKM using Protocol B were evaluated with a standard set of experiments. The iPSC colonies expressed nine key genes for pluripotency at similar levels to those in ESCs (Fig. 3A). Three genes highly expressed in MEFs — *Thy1*, *Col6a2* and *Fgf7* — were downregulated to ESC levels in iPSCs (Fig. 3B). When iPSCs were subcutaneously injected into NOD/SCID mice, they formed teratomas that contained tissues derived from all three germ layers (Fig. 3C). Furthermore, iPSCs formed chimeric mice upon aggregation with 8-cell-stage ICR mouse embryos (Fig. 3D). When one of the chimeric mice was mated with an ICR female mouse, all six pups had a brown coat color, proving that the derivatives of iPSCs contributed to germ cells (Fig. 3E).

**Figure 4:**
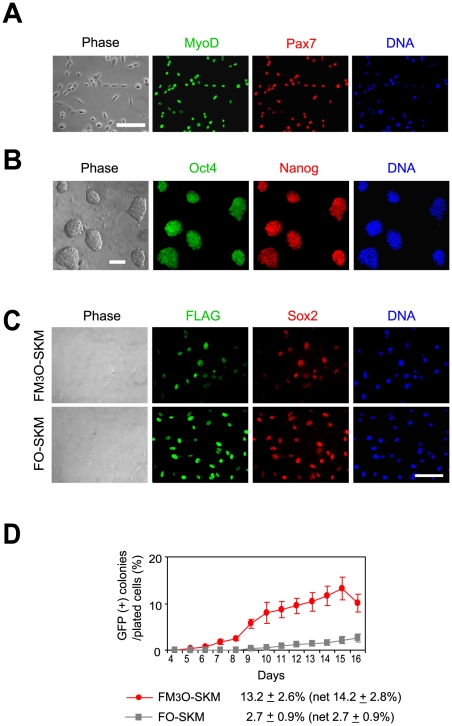
Preparation of iPSCs from mouse myoblasts with FM_3_O-SKM and KSR using Protocol B. (A) Double immunofluorescence staining of myoblasts with anti-MyoD antibody and anti-Pax7 antibodies on day 2. DNA was counterstained with Hoechst 33342. More than 98% of the cells expressed both MyoD and Pax7, confirming the high purity of myoblasts used to make iPSCs. Bar, 100 µm. (B) Double immunofluorescence staining of myoblast-derived iPSC colonies on day 30 with antibodies against Oct4 and Nanog. Because transduced *Oct4* had already been supressed by this time point, endogenous Oct4 protein was represented in this result. Bar, 100 µm. (C) Double immunofluorescence staining of myoblasts transduced with FM_3_O, FO and Sox2 on day 2. Myoblasts were stained with anti-FLAG antibody (recognizing FM_3_O and FO) and anti-Sox2 antibody. Bar, 100 µm. (D) Formation of iPSCs from myoblasts with FM_3_O-SKM and FO-SKM with Protocol B. Maximum efficiency obtained with each gene combination is shown at the bottom. The efficiency was calibrated according to the transduction efficiency and reported as net efficiency in parentheses.

To understand if the highly efficient generation of iPSCs using FM_3_O-SKM and Protocol B was specific to MEFs, we isolated myoblasts from adult Oct4-GFP mice (Fig. 4A) and prepared iPSCs from these cells (Fig. 4B). The co-transduction efficiency was 92.8±3.4% for FM_3_O-SKM and 99.0±1.5% for FO-SKM (Fig. 4C). M_3_O-SKM was slower and less efficient at inducing myoblasts into iPSCs (13.2%, net efficiency was 14.2%, Fig. 4D) as compared with MEFs; nonetheless, it was substantially more efficient than our previous result obtained with OSKM in the presence of KSR and feeder cells (0.0025%) [7].

**Figure 5:**
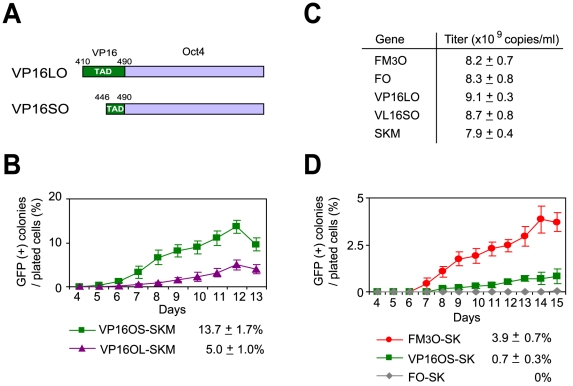
Comparison of the efficiency of making mouse iPSCs with FM_3_O, VP16SO and VP16LO using Protocol B. (A) Schematic drawings of the VP16-tagged *Oct4*s, called VL16LO and VL16SO. Numbers indicate amino acid positions of VP16. (B) Formation of iPSCs from MEFs with two VP16-Oct4 fusion genes and SKM. (C) Virus titer of each construct measured with quantitative RT-PCR. Mean ± SEM from three independent experiments is shown. (D) Formation of iPSCs from MEFs without c-*Myc*.

Because a fusion gene between *Oct4* and a fragment of the VP16 TAD (amino acids 446–490) also increased the efficiency of making iPSCs [8], we fused the same fragment and the full-length VP16 to *Oct4* (called VP16SO and VP16LO, respectively, Fig. 5A). While these two VP16 fusion genes were indeed much more efficient than wild-type *Oct4*, they were still less efficient than M_3_O (Fig. 5B). This difference was not due to a difference in viral titers (Fig. 5C). We also compared the *Oct4*-fusion genes without c-*Myc* because it causes tumor formation in vivo. Although the efficiency significantly dropped for all these fusion genes, M_3_O-SK still induced iPSCs in 3.9% of MEFs (Fig. 5D), which was more efficient than transduction of wild-type *Oct4* in the presence of *c-Myc* (FO-SKM, 2.3%, Fig. 2C).

**Figure 6:**
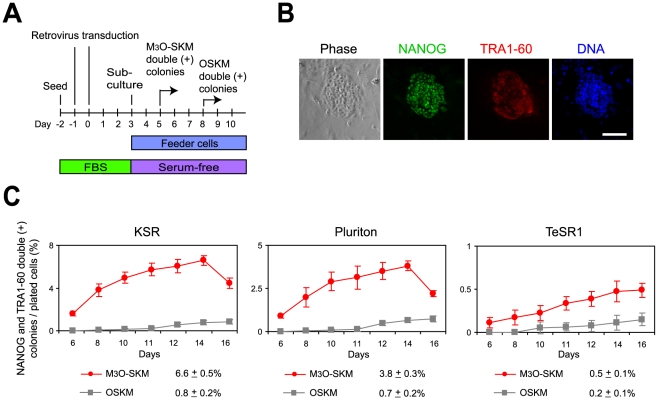
Human iPSC formation from foreskin fibroblasts transduced with M_3_O-SKM and OSKM. (A) Schematic drawing of the protocol used to make human iPSCs. (B) Double immunofluorescence staining of human iPSCs with antibodies against NANOG and TRA1-60 on day 8. DNA was counterstained with Hoechst 33342. Bar, 100 µm. (C) Temporal profiles of iPSC colony formation in three different serum-free media. Transduced fibroblasts were seeded at 2,000 cells/well of a 12-well plate on feeder cells. Immunostaining of NANOG and TRA1-60 was used to monitor iPSC colony formation. Highest efficiency is reported at bottom.

**Figure 7:**
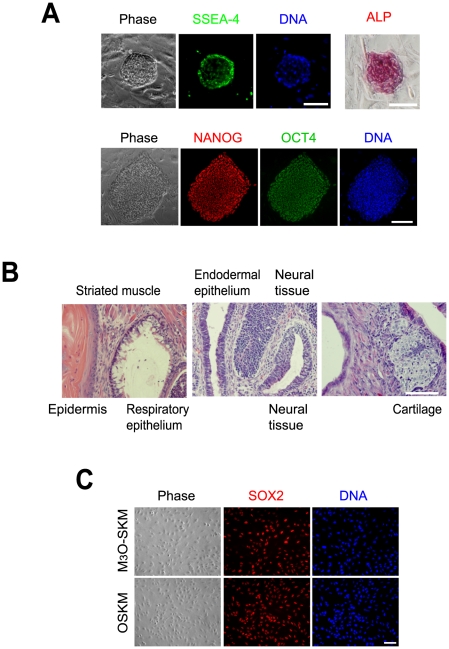
Expression of pluripotency markers in cloned human iPSCs and their ability to form teratomas. (A) Alkaline phosphatase staining and immunofluorescence staining of pluripotency markers in cloned human iPSCs obtained with M_3_O-SKM on day 27 after four passages. Bar, 100 µm. (B) Hematoxylin and eosin staining of teratoma sections derived from an iPSC clone after subcutaneous injection into an NOD/SCID mouse. Bar, 200 µm. (C) Immunofluorescence staining of foreskin fibroblasts with anti-SOX2 antibody on day 2 after transduction of M_3_O-SKM and OSKM. Bar, 100 µm.

Previously we showed that M_3_O-SKM induces iPSC formation from adult human dermal fibroblasts at an efficiency of 0.30% in feeder-free culture with KSR when seeded at 17,000 cells/well of a 12-well plate, in contrast to 0.0052% with OSKM [4]. Following the success of Protocol B as described above, we seeded transduced human neonatal fibroblasts at densities ranging from 1,000 to 17,000 cells/well of a 12-well plate on feeder cells (Fig. 6A). The number of ESC-like colonies that expressed NANOG and TRA1-60 were counted as iPSC colonies (Fig. 6B). When transduced fibroblasts were seeded on feeder cells at 17,000 cells/well, the efficiency of iPSC formation was raised to 1.7%. However, as with mouse iPSCs, the highest efficiency was obtained at 2,000 cells/well. At this cell density, iPSC colonies emerged around day 5 with M_3_O-SKM and day 8 with OSKM (Fig. 6C, KSR). The colony number peaked on day 14 with 6.6% of fibroblasts becoming iPSCs with M_3_O-SKM, in contrast to 0.8% on day 16 with OSKM. Two additional serum-free media were less effective than KSR (Fig. 6C, Pluriton and TeSR1). Cloned iPSCs prepared with M_3_O-SKM and KSR also expressed other pluripotency markers, including SSEA-4, alkaline phosphatase, NANOG and endogenous OCT4 on day 27 (Fig. 7A). Pluripotency of an iPSC clone established with M_3_O-SKM and KSR was verified with teratoma formation (Fig. 7B). In these experiments we transduced M_3_O, *OCT4*, *SOX2*, *KLF4* and c-*MYC* in separate viruses. Since KLF4 and c-MYC were endogenously expressed in fibroblasts [9], transduction efficiency of these genes could not be determined but transduction efficiency of SOX2 was sufficiently high (87.0% with M_3_O-SKM and 88.9% with O-SKM) (Fig. 7C).

In conclusion, the present study shows that the efficiency of making iPSCs can be drastically raised by changing the culture medium and the density at which transduced cells are seeded on feeder cells. A few other studies have achieved comparable efficiency of making mouse and human iPSCs. For instance, pre-B cells and myeloid progenitor cells isolated from iPSC-derived mice were converted to secondary iPSCs at an efficiency of 92% and 27.5%, respectively, after activation of the OSKM transgenes [10], [11]. Another study demonstrated around a 10% efficiency of preparing mouse and human primary iPSCs (i.e. not from iPSC-derived differentiated cells) using the *miR302/367* cluster [12]. However, to the best of our knowledge, the present study achieved the highest efficiency of making primary mouse iPSCs with an OSKM variant. Furthermore, the difference in efficiency between FM_3_O-SKM (25.8%) and FO-SKM (2.3%) underscores the importance of the transactivation capacity of Oct4 as a major limiting factor in iPSC formation.

## Materials and Methods

### Ethics Statement

All animal experiments were approved by the Institutional Animal Care and Use Committee at the University of Minnesota (1002A78174).

### Preparation of iPSCs from MEFs

The FLAG sequence DYKDDDDK was added to the amino terminus of M_3_O and Oct4 to create the FM_3_O and FO constructs in the pMXs-IP vector [13]. Full-length (amino acids 411–490) and the second half (amino acids 446–490) of the VP16 TAD were fused to the amino terminus of the mouse *Oct4* gene in the pMXs-IP vector to prepare the VP16LO and VP16SO constructs, respectively. Plat-E cells [14] were seeded at 5×10^5^ cells/3.5 cm dish on day −4. Four micrograms of the pMXs-IP vectors encoding FO, FM_3_O, VP16LO, VP16SO and polycistronic SKM were separately transfected into Plat-E cells with 10 µl Lipofectamine 2000 (Invitrogen) on day −3 to prepare virus supernatant. Virus titer was measured using a Retro-X qRT-PCR titration kit (Clontech). The medium was replaced with 1.5 ml DMEM with 10% FBS on day −2. MEFs prepared from Oct4-GFP transgenic mice [15] were seeded at 5×10^4^ cells/well of a 12-well plate on day −2 in DMEM with 10% FBS. Virus supernatant was harvested on day −1 and day 0 and filtered through a 0.45 µm syringe filter. Two hundred and fifty microliters of each virus supernatant was added to each well of MEFs with 10 µg/ml polybrene. In Protocol A, culture medium was changed to iPSC medium (DMEM, 20% KSR (Invitrogen), 100 µM MEM non-essential amino acids, 100 µM 2-mercaptoethanol, 2 mM L-glutamine and 1000 u/ml leukemia inhibitory factor) on day 1, and cells were maintained without subculture. KSR was replaced with 15% FBS only in the experiment shown in the middle set of Fig. 1D. Protocol B was used to measure the efficiency of making iPSCs. For this protocol, transduced MEFs were subcultured onto irradiated MEFs at 1,000–4,000 cells/well of a 12-well plate in iPSC medium containing KSR on day 1. The efficiency of making iPSCs was calculated each day thereafter by dividing the number of GFP-positive colonies by the seeded cell number. Net efficiency was obtained by dividing the above-mentioned efficiency of making iPSCs by the efficiency of viral co-transduction obtained with immunofluorescence staining of transduced cells.

### Preparation of iPSCs from mouse myoblasts

Satellite cell-derived myoblasts were isolated from the hind limb skeletal muscle of one-month-old adult Oct4-GFP transgenic mice as previously described [16]. Myoblasts were cultured on collagen-coated dishes in myoblast growth medium (HAM's F-10 medium supplemented with 20% FBS and 5 ng/ml basic fibroblast growth factor (R&D Systems)) and used to make iPSCs with Protocol B.

### Preparation of human iPSCs

The human M_3_O fusion gene was composed of the M_3_ domain of human *MYOD* fused to the amino terminus of full-length human *OCT4* cDNA [13]. Human M_3_O, *OCT4*, *SOX2*, *KLF4* and c-*MYC* (Addgene) were separately inserted into the pMXs-IP vector and transfected into Plat-A cells (Cell Biolabs) with Lipofectamin 2000 (Invitrogen) on day −3. On day −2, 2.7×10^4^ foreskin fibroblasts obtained from a newborn boy (Lonza) were plated in a well of a 12-well plate in DMEM with 10% FBS. Virus supernatant was harvested from Plat-A cells on day −1 and day 0, filtered through a 0.45 µm syringe filter, and transduced each day into fibroblasts with polybrane. Transduced cells were harvested on day 3 with trypsin and subcultured separately in three different media at 2×10^3^ cells/well onto irradiated MEFs in 12-well plates. The first medium (KSR medium) was composed of KnockOut DMEM/F-12 (Invitrogen), 20% KSR, 100 µM MEM non-essential amino acids, 1% insulin-transferrin-selenium (Invitrogen), 0.1 mM 2-mercaptoethanol, 1× GlutaMAX (Invitrogen) and 4 ng/ml basic FGF. Pluriton™ Reprogramming Medium (Stemgent) and mTeSR®1 Medium (STEMCELL Technologies) were prepared following the instructions provided by the manufacturers and used without additional components. Culture media were changed every other day.

### Immunofluorescence staining and alkaline phosphatase staining

iPSCs were fixed with 4% formaldehyde in phosphate buffered saline for 10 min and treated with 0.5% Triton X-100 for 3 min to permeabilize the plasma membrane. Cells were then incubated with primary and secondary antibodies for 1 hr each at 25°C using the antibodies listed in Table S1. Hoechst 33342 was used to counterstain DNA. Fluorescence photographs were taken with an AxioCam charge coupled device camera attached to an Axiovert 200 M fluorescence microscope (all from Carl Zeiss) equipped with a 10× A-Plan Ph1 Var1 objective (numerical aperture 0.25). Images were processed with Photoshop 7.0 (Adobe Systems). iPSCs were also stained with an Alkaline Phosphatase Detection Kit (Millipore SCR004) following the manufacturer's instructions.

### Quantitative RT-PCR (qRT-PCR)

iPSC colonies were individually harvested, and cDNA was directly prepared from cells with a Cells-to-cDNA II kit (Ambion). qRT-PCR was performed on a Realplex 2S system (Eppendorf) with a GoTaq qPCR Master mix (Promega). Primers are listed in Table S2. The mRNA level of each gene was normalized using the mRNA encoding glyceraldehyde 3-phosphate dehydrogenase (GAPDH). The feeder-free ES cell line CGR8 (European Collection of Cell Cultures) was used as positive control.

### Aggregation chimera and teratoma formation

Eight to 10 iPSCs were briefly exposed to acidic Tyrode's solution (Millipore) and transferred into a microdrop of KSOMaa solution (Millipore) that contained a zona-free 8-cell-stage mouse embryo of the ICR strain (albino). On the next day morula stage embryos that contained GFP-positive iPSCs were transferred into the uteri of 2.5 days post-coitum pseudopregnant recipient mice to obtain chimeric mice. Teratomas were induced by subcutaneously injecting one million iPSCs into NOD/SCID mice. After four weeks teratomas were isolated, fixed with 10% formalin, and embedded in paraffin. Histological sections (5 µm thick) were stained with haematoxylin and eosin to identify tissue types.

## Supporting Information

Table S1 **Antibodies used for immunofluorescence staining.**
(DOCX)

Table S2 **Primers used for quantitative RT-PCR.**
(DOCX)
