# Supplementary material for: Efficient iPS Cell Production with the MyoD Transactivation Domain in Serum-Free Culture
Source: PLoS One. 2012 Mar 30;7(3):e34149. doi: 10.1371/journal.pone.0034149 (PMC3316619; doi:10.1371/journal.pone.0034149)
Supplement: Table S1 — Antibodies used for immunofluorescence staining. (DOCX) [file pone.0034149.s001.docx]

**Table S1. Antibodies used for immunofluorescence staining**

**Primary antibodies**

| **Antigen** | **Manufacturer** | **Catalog #** |
| --- | --- | --- |
| FLAG  MyoD  PAX7  Oct4  Nanog  Nanog  Sox2  SSEA-1  SSEA-4, FITC-labeled  TRA-1-60, PE-labeled | Sigma Aldrich  Santa Cruz Biotechnology  Developmental Studies Hybridoma Bank  Santa Cruz Biotechnology  R&D Systems  Abcam  Santa Cruz Biotechnology  R&D Systems  BD Biosciences  BD Biosciences | F1804  sc-304  None  sc-8628  AF1997  ab21624  sc-17320  FAB2155P  560126  560193 |

**Secondary antibodies**

| **Name** | **Manufacturer** | **Catalog #** |
| --- | --- | --- |
| PE-labeled anti-mouse Ig(M+G)  Alexa Fluor 488-labeled anti-mouse IgG Alexa Fluor 488-labeled anti-rabbit IgG  Alexa Fluor 488-labeled anti-goat IgG  Alexa Fluor 555-labeled anti-mouse IgG  Alexa Fluor 555-labeled anti-rabbit IgG  DyLight488-labeled anti-goat IgG  Cy3-labeled anti-goat IgG | BD Biosciences  Invitrogen  Invitrogen  Invitrogen  Invitrogen  Invitrogen  Jackson ImmunoResearch  Jackson ImmunoResearch | 550589  A11029  A11304  A11055  A21424  A21429  805-485-180  805-165-180 |
