# Supplementary material for: Efficient iPS Cell Production with the MyoD Transactivation Domain in Serum-Free Culture
Source: PLoS One. 2012 Mar 30;7(3):e34149. doi: 10.1371/journal.pone.0034149 (PMC3316619; doi:10.1371/journal.pone.0034149)
Supplement: Table S2 — Primers used for quantitative RT-PCR. (DOCX) [file pone.0034149.s002.docx]

**Table S2. Primers used for quantitative RT-PCR**

| **Gene** | **Forward** | **Reverse** |
| --- | --- | --- |
| Oct4 endogenous | TCTTTCCACCAGGCCCCCGGCTC | TGCGGGCGGACATGGGGAGATCC |
| Sox2 endogenous | AAAGGAGAGAAGTTTGGAGCCCGA | GGGCGAAGTGCAATTGGGATGAAA |
| Nanog | AGCAGAAGATGCGGACTGTGTTCT | CCGCTTGCACTTCATCCTTTGGTT |
| Fbx15 | TTGGTGTGCATTGGATGTGTGAGC | TCAGACTTGTGGCTGTTTCCTCCA |
| Eras | ACTGCCCCTCATCAGACTGCTACT | CACTGCCTTGTACTCGGGTAGCTG |
| Rex1 | CTGGATTTCAACTTGCGCACCCAT | TTCAGCATTTCTTCCCGGCCTTTG |
| Fgf4 | GGTGAGCATCTTCGGAGTG | GTAGGATTCGTAGGCGTTGTAG |
| Tert | GAAGCAGGCCCAGCATTTC | GGATGTTTTGTCCGCTCATAGTT |
| Dppa3 | GGAAGTTCAAAGCGCCTTTCCCAA | ATGGCTCACTGTCCCGTTCAAACT |
| Thy1 | GCCTGACCCGAGAGAAGAAGAAG | TGGTGGTGAAGTTCGCTAGAGTAAG |
| Col6a2 | CCACCACTGAAAGGAACAACAA | TCCAACACGAAATACACGTTGAC |
| Fgf7 | CCATGAACAAGGAAGGGAAA | TCCGCTGTGTGTCCATTTAG |
| GAPDH | TGCACCACCAACTGCTTAG | GATGCAGGGATGATGTTC |
